# The association of low serum magnesium levels with frailty among hemodialysis patients

**DOI:** 10.1038/s41598-023-42187-x

**Published:** 2023-09-11

**Authors:** Mayuko Hori, Kaoru Yasuda, Hiroshi Takahashi, Kunio Morozumi, Shoichi Maruyama

**Affiliations:** 1 Department of Nephrology, Masuko Memorial Hospital, 35-28 Takehashi-cho, Nakamura-ku, Nagoya, Aichi 453-8566 Japan; 2 Department of Nephrology, Fujita Health University School of Medicine, Toyoake, Aichi Japan; 3 Department of Nephrology, Nagoya University Graduate School of Medicine, Showa-ku, Nagoya, Aichi Japan

**Keywords:** Renal replacement therapy, Nutrition

## Abstract

Frailty is common among hemodialysis patients and is associated with mortality and fractures. Hypomagnesemia is also known to be a risk factor for mortality and fractures and has been shown to be significantly associated with muscle performance indexes. However, little is known about the association between hypomagnesemia and frailty. We enrolled 339 outpatients who underwent hemodialysis and assessed frailty using the Clinical Frailty Scale (CFS), a 7-point subjective assessment tool based upon clinical judgment. We examined the association between serum magnesium levels and frailty evaluated using the CFS. The median CFS score was 3 points, and 49 (14.5%) patients had frailty (CFS score ≥ 5). In multiple regression analysis, serum magnesium levels were independently associated with increased CFS scores (β = − 0.126, P = 0.005) adjusted for age, body mass index, diabetes, cardiovascular diseases, prevalent fractures, serum albumin and C-reactive protein. The adjusted odds ratio for frailty was 2.85 [95% confidence interval (CI) 1.23–6.97, P = 0.014] in the lower serum magnesium group categorized based on the median value. Furthermore, with regard to model discrimination, adding serum magnesium levels to the established risk factors significantly improved net reclassification (0.520, P < 0.001) and integrated discrimination (0.023, P = 0.031). Lower serum magnesium levels may be associated with the severity and definition of frailty independent of well-known risk factors.

## Introduction

Frailty is typically defined as a loss of physiological reserves leading to increased vulnerability^1^. Frailty prevalence is high among end-stage renal disease (ESRD) patients, from 14 to 73% depending on the patient population and frailty assessment method used^2–5^. Among dialysis patients, frailty is associated with an increased risk of mortality and hospitalization^4,6^.

The Clinical Frailty Scale is an easily applicable subjective frailty assessment tool based on overall clinical impression in the domains of mobility, energy, physical activity and function^1^. Although the Fried Phenotype, a questionnaire-based, objective and combined assessment, is the most commonly used tool to evaluate frailty^7^, it is difficult to conduct measurements for all patients, especially those who have depression, paralysis or dementia, in routine clinical practice because objective measurements, including of grip strength and walking speed, are needed. Past cohort studies of dialysis patients have shown that the Clinical Frailty Scale (CFS) is associated with mortality and is not inferior to objective assessment tools because this scale realistically reflects the comprehensive clinical condition^8,9^.

The risk factors known to contribute to frailty were aging, inflammation^10^, anemia^11^, underweight or overweight^10,12,13^ and chronic diseases such as diabetes^12^, cardiovascular diseases^12,14^ and chronic obstructive pulmonary disease^12^; some of these factors are irreversible or difficult to improve with treatment. It would be important to research the other potentially modifiable factors that can be targeted with therapeutic intervention.

Magnesium (Mg) is an essential trace element that plays a key role in various cellular processes^15,16^, and nearly 30% of the magnesium in the body is found in muscle^17^. A past study showed that higher magnesium concentrations were significantly associated with indexes of muscle performance^18^, whereas severe muscle weakness, such as respiratory muscle paralysis was seen in cases of severe hypermagnesemia like above 5 mmol/L (12 mg/dL)^19^. Some past cohort studies have reported that a lower serum magnesium concentration was associated with an increased risk of fractures^20–22^, and we also reported that low serum magnesium was associated with incident fractures independent of bone mineral density among hemodialysis patients in a previous study^23^, while the association between frailty and falls or fractures has also been reported^24,25^. However, little is known about the relationship between low serum magnesium concentrations and frailty. In this study, our objective was to investigate the association between serum magnesium and frailty in hemodialysis patients.

## Results

### Patient characteristics

A total of 339 outpatients were enrolled in this study. Their mean age (± SD) was 63.8 ± 13.1 years, 76.7% were male, and 41.3% had diabetes mellitus. The median CFS score was 3 points, and 49 patients (14.5%) in this study were frail (Table 1).

**Table 1:** Patient characteristics.

	All patients n = 339	CFS score ≥ 5 n = 49	CFS score < 5 n = 290	P value
Age (years)	63.8 ± 13.1	76.4 ± 11.1	61.6 ± 12.1	< 0.001
Sex (%), male	76.7	67.4	78.3	0.10
BMI (kg/m^2^)	22.3 ± 4.4	19.8 ± 3.4	22.6 ± 4.4	< 0.001
Dialysis vintage (years)	6.9 (3.8, 13.7)	8.2 (4.7, 12.3)	6.7 (3.6, 13.8)	0.68
Serum magnesium (mg/dL)	2.5 (2.3, 2.8)	2.3 (2.1, 2.5)	2.6 (2.3, 2.8)	< 0.001
Serum Alb (g/dL)	3.6 ± 0.3	3.3 ± 0.3	3.6 ± 0.2	< 0.001
Hb (g/dL)	11.6 ± 1.2	11.6 ± 1.1	11.6 ± 1.2	0.78
CRP (mg/dL)	0.09 (0.03, 0.28)	0.27 (0.09, 0.62)	0.07 (0.03, 0.23)	< 0.001
ESRD cause				0.002
Diabetes	37.0	63.3	32.5	
GN	26.0	14.3	28.0	
Nephrosclerosis	10.1	6.1	10.7	
PKD	6.5	4.1	6.9	
Other or unknown	20.4	12.2	21.8	
Comorbidities				
Diabetes (%)	41.3	65.3	37.2	< 0.001
CVD (%)	36.3	57.1	32.8	0.001
COPD (%)	2.4	2.0	2.4	0.87
Prevalent fractures (%)	27.4	55.1	22.8	< 0.001
Clinical Frailty Scale	3 (2, 4)	5 (5, 6)	3 (2, 3)	< 0.001

Data are expressed as the means ± SDs or medians (interquartile ranges).

*BMI* body mass index, *Alb* albumin, *Hb* hemoglobin, *CRP* C-reactive protein, *GN* glomerular nephritis, *PKD* polycystic kidney disease, *CVD* cardiovascular disease, *COPD* chronic obstructive pulmonary disease.

Only one patient had the highest 7-point severe frailty score among the patients.

The patients with frailty (CFS ≥ 5) were older and had a lower body mass index (BMI), lower serum magnesium levels, lower albumin levels and higher C-reactive protein (CRP) levels; in addition, more patients had comorbidities of diabetes and cardiovascular diseases and a history of fractures among those patients with frailty.

### Serum magnesium levels and clinical frailty scale

Table 2 shows that age, comorbidity of cardiovascular diseases and diabetes, history of fractures and CRP levels were positively correlated with the CFS, and BMI, serum magnesium levels and serum albumin levels were negatively correlated with the scale in univariate models. In multivariate models that adjusted for age, BMI, diabetes, cardiovascular diseases, prevalent fractures, serum albumin, and CRP, which were variables with P < 0.05 in univariate analyses, serum magnesium levels were independently associated with increased CFS scores (β = − 0.126, P = 0.005) (Table 2).

**Table 2:** Factors affecting the Clinical Frailty Scale score: Simple and multivariate regression analyses.

Variables	Univariate		Multivariate	
	Β	P	β	P
Age (years)	0.461	< 0.001	0.235	< 0.001
Sex (%), male	− 0.098	0.070		
BMI (kg/m^2^)	− 0.200	< 0.001	− 0.138	0.003
Dialysis vintage (years)	0.087	0.10		
Serum magnesium (mg/dL)	− 0.236	< 0.001	− 0.126	0.005
Diabetes	0.308	< 0.001	0.242	< 0.001
CVD	0.262	< 0.001	0.138	0.002
COPD	0.022	0.68		
Prevalent fractures	0.271	< 0.001	0.111	0.034
Serum Alb (g/dL)	− 0.372	< 0.001	− 0.199	< 0.001
Hb (g/dL)	− 0.011	0.82		
CRP (mg/dL)	0.223	< 0.001	0.094	0.034

*β* standardized regression coefficient, *BMI* body mass index, *CVD* cardiovascular disease, *COPD* chronic obstructive pulmonary disease, *Alb* albumin, *Hb* hemoglobin, *CRP* C-reactive protein.

In logistic regression analyses, lower serum magnesium levels (≤ median of 2.5 mg/dL) were also independently associated with frailty (adjusted odds ratio 2.85, 95% CI 1.23–6.97, P = 0.014) after adjusting for the established risk factors (Table 3).

**Table 3:** The effect of variables on frailty (Clinical Frailty Scale score ≥ 5).

Variables	Univariate		Multivariate	
	OR (95% CI)	P value	OR (95% CI)	P value
Age (years)	1.11 (1.08–1.15)	< 0.001	1.08 (1.04–1.13)	< 0.001
Sex (%), male	0.57 (0.29–1.12)	0.10		
BMI (kg/m^2^)	0.80 (0.71–0.88)	< 0.001	0.82 (0.70–0.95)	0.006
Dialysis vintage (years)	0.99 (0.95–1.03)	0.85		
Lower serum Mg (≤ 2.5 mg/dL)	3.44 (1.77–7.13)	< 0.001	2.85 (1.23–6.97)	0.014
Diabetes	3.17 (1.70–6.09)	< 0.001	4.25 (1.84–10.48)	< 0.001
CVD	2.73 (1.48–5.12)	0.001	3.10 (1.36–7.39)	0.006
COPD	0.84 (0.04–4.88)	0.87		
Prevalent fractures	4.16 (2.23–7.85)	< 0.001	2.06 (0.91–4.62)	0.079
Serum Alb (g/dL)	0.05 (0.01–0.14)	< 0.001	0.19 (0.04–0.85)	0.03
Hb (g/dL)	0.99 (0.76–1.27)	0.97		
CRP (mg/dL)	1.92 (1.23–3.02)	0.004	1.09 (0.58–2.03)	0.76

*OR* odds ratio, *CI* confidence interval, *Mg* magnesium, *BMI* body mass index, *CVD* cardiovascular disease, *COPD* chronic obstructive pulmonary disease, *Alb* albumin, *Hb* hemoglobin, *CRP* C-reactive protein.

Regarding model discrimination, the addition of lower serum magnesium levels to the diagnostic model based on established risk factors with P < 0.05 in univariate analyses (age, BMI, serum albumin, CRP, comorbidity of diabetes and cardiovascular diseases and prevalent fractures) significantly improved the NRI (0.520, P < 0.001) and the IDI (0.023, P = 0.031). The C-index also improved (from 0.897 to 0.904) but did not reach significance (P = 0.26) (Table 4).

**Table 4:** Discriminatory ability of each prediction model for frailty based on the C-index, net reclassification improvement (NRI), and integrated discrimination improvement (IDI).

Variables	C-index (95% CI)	P value	NRI	P	IDI	P value
Established risk factors	0.897 (0.844–0.951)	Reference		Reference		Reference
Established risk factors + lower serum Mg	0.904 (0.853–0.956)	0.26	0.520	< 0.001	0.023	0.031

Established risk factors included age, BMI, comorbidities of diabetes and cardiovascular diseases, history of fractures, serum Alb and CRP as covariates with P < 0.05 in univariate logistic regression analyses.

## Discussion

In this cross-sectional study, we showed that serum magnesium levels were inversely associated with the severity of frailty. To our knowledge, this is the first study to demonstrate the association between serum magnesium levels and frailty among hemodialysis patients.

Past studies have shown poor survival and cardiovascular mortality associated with magnesium deficiency in dialysis patients^26,27^. A past cohort study of hemodialysis patients found that lower serum magnesium levels were associated with a high risk of fractures^22^, and our previous study showed that the association between serum magnesium and fractures was independent of bone mineral density^23^. There may be a possible mechanism underlying the association of lower serum magnesium with fracture or mortality. A past study showed an independent association between serum magnesium concentration and muscle performance^18^, and a study of postmenopausal women showed that an adequate magnesium concentration seems to be necessary for muscle performance^28^. A past animal study showed that magnesium depletion causes structural damage in muscle tissues by increasing oxidative stress and impaired intracellular calcium homeostasis^29^. We hypothesized that low serum magnesium may be associated with frailty through its effect on muscle weakness, which may subsequently lead to fractures or mortality. Our results shown in this study were consistent with this hypothesis. Serum magnesium might be a possible therapeutic target to prevent frailty. Regarding excess magnesium levels, although severe hypermagnesemia above 5 mmol/L (12 mg/dL) is known to cause severe muscle weakness, the highest serum magnesium level in our patients was 3.8 mg/dL. Within such a clinically relevant range, higher magnesium levels were inversely associated with the severity of frailty.

In this study, 14.5% of the hemodialysis patients were frail, which was a lower prevalence of frailty than those reported in prior studies of end-stage renal disease (ESRD) patients^2–4^, which could be because the eligible patients in our study were outpatients. In a previous cohort study of outpatients undergoing hemodialysis, the prevalence of frailty based upon CFS was 17.8%, similar to that found in our study. In our patients, the average age was 63.8 years old, and the proportion of male patients was 76.7%; thus, the patients were slightly younger and more likely to be male than prevalent dialysis patients in Japan (average age 67.5 years; male 63.8%) though likely similar to them. The proportion of diabetes as the cause of ESRD in our patients was also similar to that of prevalent dialysis patients in Japan (37.0% vs. 38.1%)^30^. Because of the nature of an unmatched retrospective cohort study, this study is susceptible to selection bias; hence, our patients likely reflect dialysis patients in Japan. The factors associated with the increased frailty levels in our patients included older age, low BMI, low serum albumin, high CRP, the comorbidity of diabetes and cardiovascular diseases, and the history of fractures, which were consistent with prior studies^10,12,31,32^. We demonstrated that combining serum magnesium levels with these well-known risk factors could increase the diagnostic ability for frailty.

Although lower serum magnesium may be associated with frailty risk partly through its relationship with comorbidity of cardiovascular diseases or prevalent fractures, the association between serum magnesium concentrations and frailty observed in our data remained significant after adjustment for the comorbidity of cardiovascular diseases and prevalent fractures.

In some studies, magnesium deficiency has been reported to be associated with malnutrition, inflammation, diabetes mellitus and cardiovascular diseases^17,33–35^, and these conditions are also known to be etiologic factors in protein-energy wasting (PEW)^36^. PEW is a state of metabolic and nutritional derangements in CKD patients, leading to loss of muscle and cachexia^37^, and frailty is closely related to PEW among CKD and HD patients^38,39^. In our data, the CFS was significantly associated with the markers of nutrition (BMI and serum albumin), the marker of inflammation (CRP) and comorbidity of diabetes and cardiovascular diseases, which were the etiologic factors of PEW, as we mentioned above. The association between frailty and low serum magnesium concentrations shown in our study may reflect the complication of PEW that might have been aggravated by magnesium depletion. However, the association remained significant after adjusting for BMI, serum albumin, CRP and comorbidity of diabetes and cardiovascular diseases.

Our study had some limitations. First, the study was conducted at a single center in Japan. Second, the association between frailty and serum magnesium does not imply causality in either direction due to the cross-sectional nature of the study. A longitudinal study may be needed to explore the relationship between serum magnesium concentration and the development of frailty. Third, in our patients, there was only one patient with severe frailty (indicated by a score of 7 points). Our results might be insufficient to detect an effect of serum magnesium on patients with very advanced frailty. Hence, the identification of frail patients who still have not reached terminal frailty would be important because they may have the possibility of preventing the progression of frailty with some therapeutic interventions. The other limitation is the limited reproducibility of CFS due to the nature of the subjective assessment.

In conclusion, the results of our study showed an independent relationship between serum magnesium levels and frailty. A possible reason for this association might be the complex relation of magnesium with muscle function, the occurrence of cardiovascular diseases, fractures and malnutrition. Further longitudinal research is needed to investigate whether serum magnesium levels are associated with the development of frailty.

## Materials and methods

### Study population

This cross-sectional study was conducted at Masuko Memorial Hospital in April 2022. The inclusion criteria were outpatient hemodialysis, age ≥ 20 years and hemodialysis treatment for at least 3 months, and the exclusion criteria included (1) unwillingness to provide consent for the retrospective analysis of the data and (2) admission to the hospital within 1 month prior to the evaluation.

All patients provided written informed consent to be involved in this study. The study protocol was conducted in accordance with the Declaration of Helsinki. The hospital’s institutional review board approved this protocol (Ethics approval number: MR4-26).

### Frailty

We used the original version of the CFS^1^ to evaluate the degree of frailty in all eligible hemodialysis patients. The original version of the scale is scored from 1 to 7 (1, very fit; 2, well without active disease; 3, well with treated comorbid disease; 4, apparently vulnerable; 5, mildly frail; 6, moderately frail; 7, severely frail), with a score of 5 or greater indicating clinical frailty (Supplementary Table S1 online)^1,40^. The nurses of the hemodialysis center were provided information about each level of frailty in CFS before study initiation and the nurse who had the most clinical information about the patients determined the CFS scores during the dialysis session in April 2022.

### Characteristics

The following data were collected: demographics (age, sex, BMI and hemodialysis vintage), the primary cause of kidney disease, comorbidity conditions (cardiovascular diseases: myocardial infarction, cerebrovascular diseases and congestive heart failure; chronic obstructive pulmonary disease; diabetes mellitus), history of fragility fractures and laboratory measurement (serum magnesium, albumin, hemoglobin and CRP) from medical records in April 2022. Blood samples were drawn from the arteriovenous fistula and the arteriovenous graft just before the first weekly hemodialysis session. The dialysate magnesium concentration was 1.0 mEq/L.

### Statistical analyses

Continuous variables are expressed as the means ± standard deviations (SDs) or medians with interquartile ranges (IQRs). For comparisons between two groups, Student’s *t* test or the Mann–Whitney *U* test was used for continuous variables, and the Chi-square test was used for categorial variables. Simple and multiple regression analyses were performed to identify the independent factors for CFS scores. We divided the patients into two groups based on the median serum magnesium levels and evaluated the effects of lower serum magnesium levels and the other variables on frailty (CFS ≥ 5) using logistic regression analysis. All variables with P < 0.05 in univariate analyses were entered into a multivariate model. We evaluated the diagnostic ability of the variables for frailty using receiver operating characteristic (ROC) curves and calculated the C-index, net reclassification improvement (NRI) and integrated discrimination improvement (IDI) to assess whether the diagnostic ability for frailty was improved after the addition of lower serum magnesium levels to the baseline model with established risk factors. The C-index was defined as the area under the ROC curves for individual diagnostic probabilities for frailty and was compared between the two diagnostic models^41^. The NRI indicates for how many patients diagnostic probabilities for frailty were improved, while the IDI represents the average improvement in diagnostic probabilities for frailty after adding variables into the baseline model^42^. We considered P values < 0.05 to be statistically significant. All analyses were performed using JMP11 software (SAS Institute Inc., Cary, NC, USA) and R version 3.4.1.

### Ethical approval

All procedures performed in studies involving human participants were in accordance with the ethical standards of the institutional and/or national research committee and with the 1964 Helsinki Declaration and its later amendments or comparable ethical standards. This study was approved by the Masuko Memorial Hospital Ethics Committee (Ethics approval number: MR4-26).

### Informed consent

All patients provided written informed consent to be involved in this study.

### Supplementary Information

Supplementary Table S1.

## Data Availability

The data collected for this study cannot be shared publicly because they contain information that could compromise the privacy of the research participants. The data are available from the corresponding author upon request.
