# Supplementary material for: The association of low serum magnesium levels with frailty among hemodialysis patients
Source: Sci Rep. 2023 Sep 11;13:14982. doi: 10.1038/s41598-023-42187-x (PMC10495433; doi:10.1038/s41598-023-42187-x)
Supplement: Supplementary file 1 — Supplementary Table S1. [file 41598_2023_42187_MOESM1_ESM.pdf]

Supplementary Table S1. The Canadian Society of Health and Aging Clinical Frailty Scale (CFS)

**CFS score**	**Interpretation**
1	Very fit: robust, active, energetic, well-motivated, and fit; fittest in their age group
2	Well: without active disease but not as fit as those in category 1
3	Well: with treated comorbid disease
4	Apparently vulnerable: not dependent but has symptoms from comorbid disease (such as being slowed up)
5	Mildly frail: limited dependence on others for instrumental activities of daily living
6	Moderately frail: help is needed for instrumental activities of daily living and activities of daily living
7	Severely frail: completely dependent on others for instrumental activities of daily living and activities of daily living or terminally ill
